# Impacts of Habit Formation Effect on Food Consumption and Nutrient Intake in Rural China

**DOI:** 10.3390/nu16040505

**Published:** 2024-02-10

**Authors:** Jinshang Wen, Wenbo Zhu, Xinru Han, Xiudong Wang

**Affiliations:** 1 Institute of Agricultural Economics and Development, Chinese Academy of Agricultural Sciences, Beijing 100081, China; wenjinshang@caas.cn (J.W.); hanxinru@caas.cn (X.H.); 2 Rural Development Institute, Chinese Academy of Social Sciences, Beijing 100732, China; zhuwenbo@cass.org.cn; 3 Center for Strategic Studies, Chinese Academy of Agricultural Sciences, Beijing 100081, China; 4 Chinese Institute of Agricultural Development Strategies, Beijing 100081, China

**Keywords:** habit formation effect, food consumption, nutrient intake, income elasticity, Chinese rural residents, dynamic AIDS model

## Abstract

This study employs panel data and a dynamic Almost Ideal Demand System (AIDS) model to investigate the habit formation effect of food consumption among Chinese rural residents and its consequential impact on nutritional intake. The dataset, spanning from 2012 to 2018, encompasses nine provinces in China and involves 5390 rural households. The findings reveal that, excluding beef, mutton, and poultry, there are significant habit formation effect on the consumption of food categories, notably grains, vegetables, and edible oils. Lower-income and younger demographics demonstrate a more pronounced reliance on established dietary habits. Influenced by the habit formation effect, there is a substantial reduction in the income elasticity differences across various food types. Overlooking the habit formation effect in food consumption would lead to an underestimation of the income elasticity of energy, fat, and carbohydrates. This suggests that, over the long term, food consumption habit formation is a pivotal factor in enabling the enhancement of residents’ dietary structures, amplifying the incremental energy intake associated with income increases, and accelerating the transition towards nutritional surplus. The conclusions drawn from this study offer valuable insights for ensuring food security and nutritional balance. Policy-makers of food and nutrition strategies should duly consider the habit formation effect on residents’ food consumption, and seek to optimize dietary patterns and promote nutritional transformation by food consumption habit intervention.

## 1. Introduction

Amid rapid socioeconomic growth and improvement in living standards, the dietary structure in China has undergone significant shifts. Staple food consumption, including grains and edible oil, has stabilized, while vegetable intake has slightly grown, and the consumption of animal-based products, including meat, poultry, egg, dairy, and aquatic products, continues to rise [1,2,3]. According to the National Bureau of Statistics of China (NBSC), from 2013 to 2021, per capita consumption of vegetable and fruit increased by 12.6% and 49.9% respectively, whereas edible oil saw a minor rise of 1.9% and grains declined by 2.8%. Animal-based food consumption per capita also surged, with meats, poultry, eggs, dairy, and aquatic products increasing by 28.5%, 70.8%, 61.0%, 23.1%, and 36.5%, respectively.

However, this transition towards a higher intake of animal-sourced proteins and fats, coupled with a decline in physical activity, has sparked concerns over nutritional imbalances and an associated rise in diet-related health risks such as overweight and obesity [4,5]. As of 2018, the average Body Mass Index (BMI) of Chinese residents exceeded the overweight threshold, with an alarming 34.3% and 16.4% prevalence of overweight and obesity in adults, respectively [6,7]. This suggests that over half of the population falls under the overweight or obese category, highlighting the pressing need to address these emerging health challenges.

Influences on food consumption behaviors among residents extend beyond income and price to include entrenched dietary habits, especially in regions with strong local taste preferences like rural China [8,9,10]. These habits, influenced by local food resources and dietary culture, create stable dietary patterns and period-over-period impacts on food consumption. Neglecting these habitual influences may bias food and nutrition consumption analyses, and then impact public health policy formulation [11,12]. Thus, this paper focuses on rural residents in China to explore the characteristics of the habit formation effect in food consumption and impacts on nutritional intake.

The concept of habit formation in consumption decisions was first proposed by Duesenberry, suggesting that current consumption levels are influenced by not only current income but also past consumption behavior [13]. Some researchers have devised consumption function models incorporating habit formation, thereby investigating the influence of consumption habit on individual consumption behavior [14,15,16,17]. Later scholars developed this theory and demonstrated significant habit formation effect on food consumption [18,19]. For instance, Zhen et al. [12] and Zheng et al. [20] studied the habit formation effect in non-alcoholic beverage and tobacco consumption among U.S. residents, finding significant habitual tendencies. Zhai et al. confirmed similar effects in sugar-rich food consumption among urban Chinese residents [11]. Moreover, research indicated that habit formation helps to develop healthier eating habits [21,22]. Lin et al. observed that cues of healthy eating habits can help individuals resist unhealthy foods [21], while Salvy et al. demonstrated that habit formation can improve dietary and activity behaviors in low-income preschool children and their mothers, reducing the obesity risk [22].

A thorough review of food consumption habit formation and related nutritional health issues reveals three significant gaps in the existing literature. Firstly, despite abundant international research on food consumption habit formation, there is a lack of in-depth analysis specifically focused on rural residents in China [23]. Existing studies often treat food consumption as a whole, rather than investigating differences in habit formation across food types, demographic groups, and time periods. Secondly, research methodology has been limited, with most studies adopting linear single-equation models like the double-logarithm model, while theory-based demand system models have been underused due to model complexity and data limitations. Lastly, the impact of food consumption habits on nutritional intake is seldom considered in studies, potentially biasing conclusions on nutritional health drawn from conventional food demand system models.

This paper, utilizing data from the Survey for Agriculture and Village Economy (SAVE) in rural China, constructs an AIDS model with dynamic mechanisms to analyze the habit formation effect of food consumption and its impact on nutritional intake in rural China. This study’s contributions are threefold: It explores food consumption habit formation among rural residents in China by considering food types, population groups, and temporal aspects, thereby extending the research scope within China. Using a dynamic demand system model, it rectifies biases in previously food demand elasticity estimates due to ignoring food consumption habits, providing more accurate support for food consumption demand forecasting and food security policy in China. Furthermore, it examines the impact of food consumption habits on the nutritional intake status of rural residents in China, producing insights valuable for public health policy formulation aimed at improving nutritional structures.

## 2. Materials and Methods

### 2.1. Study Design

This paper employs the dynamic demand theory introduced by Spinnewyn [24] and the Almost Ideal Demand System (AIDS) model devised by Deaton and Muellbauer [25]. By developing a dynamic AIDS model, it scrutinizes the habit formation effect for various food categories in rural China. Using the model’s estimated parameters, the study then computes the demand elasticity for each type of food and nutrients under the context of habit formation. These findings are compared with outcomes from a static model lacking the habit formation effect, thereby facilitating analysis of the impact of habit formation on both food consumption and nutritional intake.

### 2.2. Dynamic AIDS Model

#### 2.2.1. Model Specification

As per the theory of habit formation, consumer utility is derived not solely from the present consumption but also hinges on the accumulated stock of habitual consumption. In essence, utility is predominantly harvested from the consumption that surpasses previous levels [13,26,27,28]. The consumption of food i that produces utility can be expressed as $Q_{it}$ due to the level of consumption in the previous period [17]:

(1) $$Q_{it}=q_{it}-\emptyset_{i}q_{it-1}$$

Among them, the consumption of food i in the period t is described as $q_{it}$; $\emptyset_{i}$ represents the parameter for habit formation effect, measuring the impact of a unit change in the consumption of food i in the previous period on the utility consumption in the current period. $0\leq \emptyset_{i}<1$ and the larger it is, the stronger the degree of habit formation.

The budget share equation of the dynamic AIDS model is:

(2) $${\hat{w}}_{it}\left({\hat{p}}_{it},{\hat{x}}_{t}\right)=\alpha_{i}+\sum_{j}\gamma_{ij}ln{\hat{p}}_{jt}+\beta_{i}ln\frac{{\hat{x}}_{t}}{a\left({\hat{p}}_{t}\right)}+\varepsilon_{it}$$

The real price of the food j for the period t is ${\hat{p}}_{jt}=p_{jt}\left(1+r_{t}\right)/\left(1+r_{t}-\emptyset_{j}\right)$. Among them, $p_{jt}$ and $r_{t}$ are the market price of the food j for the period t and the real interest rate for the period t. ${\hat{x}}_{t}=\sum_{i=1}^{n}{\hat{p}}_{it}Q_{it}$ is the total food expenditure and ${\hat{w}}_{it}={\hat{p}}_{it}\left(q_{it}-\emptyset_{i}q_{it-1}\right)/{\hat{x}}_{t}$ is the food budget share of the food i considering the habit formation effect. $a\left({\hat{p}}_{t}\right)$ is a food price index and $lna\left({\hat{p}}_{t}\right)=\alpha_{0}+\sum_{i=1}^{n}\alpha_{i}ln{\hat{p}}_{it}+0.5*\sum_{i=1}^{n}\sum_{j=1}^{n}\gamma_{ij}ln{\hat{p}}_{it}ln{\hat{p}}_{jt}$. Additionally, $\alpha_{i}$, $\gamma_{ij}$ and $\beta_{i}$ are the parameters to be estimated and three constraints from demand theory need to be applied to them, wherein through aggregation: $\sum_{i}\alpha_{i}=1$, $\sum_{i}r_{ij}=0$, $\sum_{i}\beta_{i}=0$; homogeneity: $\sum_{j}r_{ij}=0$; symmetry: $r_{ij}=r_{ji}$.

By implementing parameter constraints, the demand system model enables the allocation of consumption expenditures within a unified system. This accurately mirrors consumer preferences, aligning with the principles of consumer choice, thus providing a holistic portrayal of consumer behavior. Given the influence of individual characteristic heterogeneity on food consumption behavior, the dynamic AIDS model incorporates variables such as household size, the ratio of the working population, age, and education level. In this study, these variables are integrated into the constant term of the dynamic AIDS model using a linear embedding technique.

In this study, we employ per capita household income and its square as instrumental variables associated with food expenditure. This approach enables us to examine and rectify the issue of endogeneity inherent in food expenditure elements and this method is supported by numerous studies [29,30,31,32]. All parameter estimations were conducted using the STATA 14 software.

#### 2.2.2. Censoring Problem

In instances where some households do not purchase all food items, we encounter an issue known as censoring. In empirical studies, zero consumption carries considerable econometric and economic implications. Given that the data utilized in this research were collected via household surveys, zero values in the consumption of specific foods are a common occurrence. Apart from grain and edible oil, the proportion of zero consumption for other food items in the sample data exceeds 5%, signifying a pronounced issue of zero consumption. Furthermore, statistical estimation procedures that fail to account for these zero observations in the dependent variable can lead to biased and inconsistent estimates of parameters [33,34]. To address this censoring problem appropriately, we employ the two-step consistent estimation method developed by Shonkwiler and Yen in the second stage of our analysis [35,36,37].

#### 2.2.3. Elasticity

Taking habit formation into account, the expenditure elasticity of food i is defined as the elasticity of the utility consumption quantity $Q_{it}$ with respect to the adjusted food expenditure ${\hat{x}}_{t}$. Once the problem of censoring is addressed, the equation for calculating food expenditure elasticity is as follows:

(3) $$e_{i}=\Phi_{it}\left(\beta_{i}/{\hat{w}}_{it}\right)+\varphi_{it}c_{i}\left({\hat{w}}_{it}-\delta_{i}z_{it}^{\prime }\tau_{i}\right)/{\hat{w}}_{it}+1$$

In Equation (3), $z_{it}$ and $\tau_{i}$ represent the vector of explanatory variables and the corresponding parameter vector, respectively, from the first step of the two-step consistent estimation model. $\varphi_{it}\left(z_{it}^{\prime }\tau_{i}\right)$ and $\Phi_{it}\left(z_{it}^{\prime }\tau_{i}\right)$ designate the probability density function and cumulative distribution function of the standard normal distribution, which are both computed in the first step of the model. $c_{i}$ refers to the coefficient of the logarithm of total food expenditure in the i-th equation, whereas $\delta_{i}$ signifies the coefficient of the cumulative distribution function ($\varphi_{it}$) in the second step.

The objective of this paper is to calculate the income elasticity of demand for various foods and nutrients within the framework of habit formation, thereby providing a precise reflection of the impact of income growth on food consumption and nutrient intake. Assuming that consumer preferences for food and other non-food items are weakly separable, we can examine food consumption behavior by constructing a two-stage budget model. The first stage scrutinizes how consumers distribute their income between food and other goods, wherein food expenditure can be expressed as a function of the food price index, household income, and other exogenous variables. The second stage then allocates the total food expenditure to various categories of food, using the dynamic AIDS model discussed above. In the first stage, we apply an expanded version of the Engel’s model [38,39]:

(4) $${\hat{w}}_{ft}=m_{0}+m_{1}lny_{t}+m_{2}{\left(lny_{t}\right)}^{2}+m_{3}ln{\hat{P}}_{ft}+b^{\prime }Z+\zeta$$

In Equation (4), ${\hat{w}}_{ft}$ represents the fraction of food expenditure ${\hat{x}}_{t}$ over household income $y_{t}$ and ${\hat{w}}_{ft}={\hat{x}}_{t}/y_{t}$; $ln{\hat{P}}_{ft}=\sum_{i=1}^{10}{\hat{w}}_{it}ln{\hat{p}}_{it}$ refers to the deflated food price index; Z denotes a vector of household characteristics, which aligns with the feature variables in the second stage of the dynamic AIDS model; b represents the corresponding parameter vector; ζ is a random error term, assumed to follow a normal distribution. Based on Equation (4), we can derive the formula for calculating the income elasticity of food expenditure:

(5) $$e_{f}=1+m_{1}/{\hat{w}}_{f}+2m_{2}lny/{\hat{w}}_{f}$$

Based on Equations (3) and (5), the income elasticity (unconditional elasticity of expenditure) of food i can be derived:

(6) $$e_{i}^{u}=e_{f}\cdot e_{i}$$

According to Huang, the indirect method was used to further calculate the elasticity of nutrient intake [40,41,42]. The income elasticity of demand for nutrient k is:

(7) $$\rho_{k}=\sum_{i=1}^{10}\frac{e_{i}^{u}a_{ki}q_{i}}{h_{k}}$$

In Equation (7), k denotes the category of nutrient, with possible values of 1, 2, 3, or 4; $h_{k}$ symbolizes the total intake of nutrient k; $a_{ki}$ signifies the average amount of nutrient k contained in a unit of food i; $q_{i}$ refers to the consumption quantity of food i; $e_{i}^{u}$ represents the income elasticity of food i, as computed from Equation (6).

### 2.3. Data and Variables

#### 2.3.1. Data Collection

**Figure 1:**
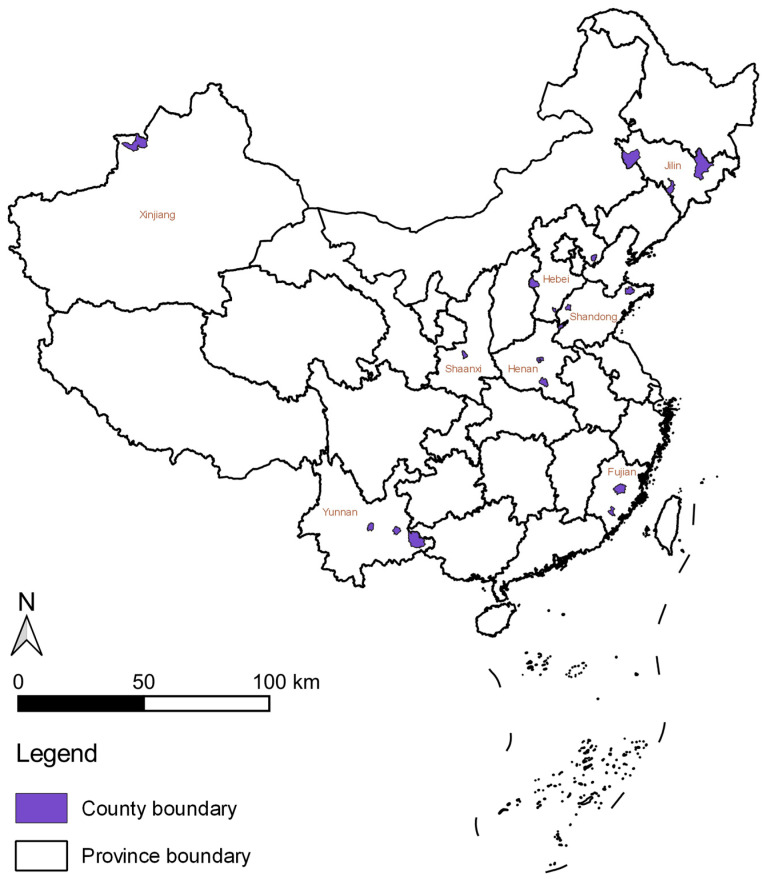
The geographical location of the study areas.

This study utilizes the Survey for Agriculture and Village Economy (SAVE) data, curated by the Institute of Agricultural Economics and Development at the Chinese Academy of Agricultural Sciences. The geographical location of the study areas is shown in Figure 1. The aim is to examine the impact of habit formation on the food consumption of rural residents at the household level [43,44,45,46]. The data, gathered annually since 2012, span six significant regions across China: North China, Northeast China, East China, Central South China, Southwest China, and Northwest China. The dataset encompasses eight provinces, including Hebei, Henan, Fujian, Jilin, Shaanxi, Yunnan, Shandong, and Xinjiang Uygur Autonomous Region, covering 28 counties (cities, districts) and 117 administrative villages. As the dynamic AIDS model incorporates lagged variables, only household samples surveyed in successive years are considered. Ultimately, this study employs a balanced panel dataset, comprising 647 valid samples per annum from 2012 to 2018, yielding a total sample size of 4529.

The SAVE dataset is segmented into four sections: basic household demographics, household production, household income and expenditure and other status. The production status section documents the consumption of self-produced food, while the income and expenditure section records the purchasing pattern of various food types. The primary focus of this paper is to analyze the consumption patterns of ten categories of food among Chinese rural residents: grains, legumes, vegetables, fruits, pork, beef and mutton, poultry, aquatic products, eggs, and edible oils. For samples with missing price data, a hierarchical approach is employed to supplement the missing values using average prices at the village, town, or county levels. By considering the nutrient content per unit of food and the proportion of edible parts, the consumption data collected in the survey can be converted into nutrient intake (as per the China Food Composition Table). The paper chiefly explores the impact of the food consumption habit on the intake of energy, protein, fat, and carbohydrates.

#### 2.3.2. Major Variables and Statistical Analysis

Drawing on the Survey for Agriculture and Village Economy (SAVE) data spanning 2012 to 2018, the evolution of food purchasing patterns in Chinese rural households is outlined in Table 1. Furthermore, there has been a marked increase in the purchase volumes of legumes, vegetables, beef and mutton, and eggs among Chinese rural households, with the increase in beef and mutton purchases surpassing 50%. This trend underscores a dietary transition towards high-quality food categories that are generally higher priced. The shifts in the purchasing volumes for fruits, pork, aquatic products, and edible oils are comparatively modest, attesting to a relatively stable consumption of these food items. Conversely, the purchasing volumes for grains and poultry have seen a substantial decrease, which can be attributed to increased food diversity offsetting some staple food consumption. Additionally, a large part of grain consumption in rural areas is sourced from home production, which is not incorporated into the purchasing data. Correspondingly, the increase in red meat consumption has precipitated a decline in poultry purchases.

According to the fluctuations in food purchasing volumes of Chinese rural households from 2012 to 2018, Figure 2 presents changes in the energy contribution ratios of the three macronutrients. Between 2012 and 2018, the energy contribution ratios of fats and carbohydrates in Chinese rural households underwent significant shifts, increasing and decreasing by 3.42 and 3.54 percentage points, respectively. The fat energy contribution ratio rose from 34.25% in 2012 to 37.68% in 2018, while the carbohydrate energy contribution ratio diminished from 54.92% in 2012 to 51.39% in 2018. The protein energy contribution ratio remained stable relatively, marginally increasing from 10.82% to 10.94%, an increment of 0.11 percentage points. Comparing these figures with the *China Food and Nutrition Development Outline (2014–2020)* and the *Chinese Dietary Guidelines (2022)*, it becomes evident that the fat energy contribution ratio in Chinese rural households consistently exceeds the stipulated 30% upper limit and is on an upward trajectory. This may potentially lead to a swift increase in overweight and obesity rates in rural areas over the short term.

Table 2 defines and provides descriptive statistics for variables related to household income and expenditure, price, and demographics. The average income of the respondent’s family stands at 45,015.98 yuan, with the average food purchase expenditure being 4660.04 yuan. This relatively low proportion of expenditure on food purchasing can be largely attributed to a significant fraction of food being sourced from self-production and self-consumption. The price statistics for various food types reveal that beef and mutton command the highest average price, at 66.45 yuan per kilogram, which is 2.89, 3.42, and 4.13 times the prices of pork, poultry, and aquatic products, respectively. The price levels of grains, vegetables, and fruits are considerably lower, aligning with market price disparities. Besides variables pertaining to income, expenditure, and price, demographic control variables have also been included in the analysis. The average family size of respondents is approximately 3.57, with the majority of household heads being male. The mean age of the household head is 51.08 years, with an average educational attainment of 8.03 years. The labor force makes up about 67% of the family. The samples from the eastern, central, and western regions account for 53%, 33%, and 13%, respectively. The sample years range from 2012 to 2018, with a constant sample size maintained annually.

## 3. Results

### 3.1. Model Estimation Results

The results for the habit formation parameters derived from the dynamic Almost Ideal Demand System (AIDS) model are presented in Table 3. The coefficient associated with the previous consumption volume ($q_{it-1}$) serves as the habit formation parameter, quantifying the effect of each additional unit of food consumed in the previous period on the utility consumption in the current period. As per our estimations, excluding beef, mutton, and poultry, the remaining eight types of food exhibited significant habit formation effect. The sequence of various food types, ranked from strong to weak habit formation, was as follows: edible oil, grains, vegetables, legumes, fruits, aquatic products, eggs, and pork. Neither beef, mutton, nor poultry exhibited noticeable habit formation characteristics. The whole estimation results are shown in Table A1.

It is discernable that Chinese rural residents maintain strong consumption habits for essential sustenance foods such as grains, vegetables, and edible oil. The volume of consumption in the current period, which generates utility, is substantially influenced by the prior period’s consumption. To reach a specific utility level, a more substantial increase in current food consumption is necessary. Conversely, the habit formation degree of animal foods is lower, signifying that the consumption habits of Chinese rural households, predominantly reliant on plant-based foods, have not undergone fundamental changes.

As time progresses, the external food consumption environment and the inherent food consumption concept of Chinese rural residents have been gradually evolving. This raises the question: do these characteristics of habit formation in food consumption also exhibit significant inter-temporal variations? Which food consumption habits have been amplified, and which have diminished? We addressed these questions by segmenting our sample data from 2012 to 2018 into two intervals, 2012–2015 and 2015–2018, and apply the same regression methodology to each to explore the inter-temporal shifts in the degree of food consumption’ habit formation.

**Figure 3:**
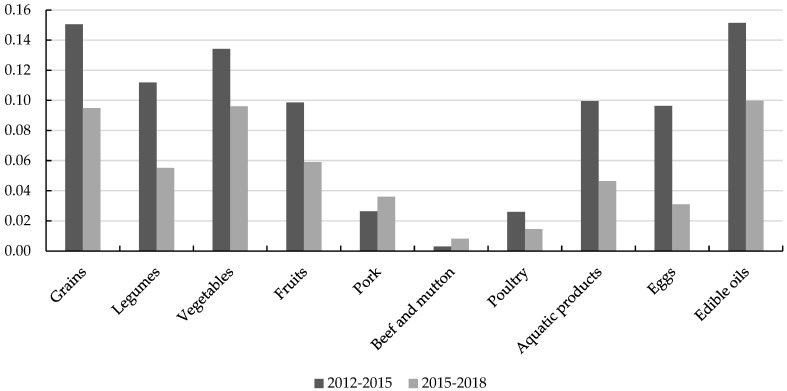
Inter-temporal variations in the habit formation effect on food consumption.

Figure 3 illustrates the variations in habit formation parameters for different types of food across the two phases. Considering the changes in habit formation parameters that passed the significance tests, the intensity of habit formation for grains, legumes, vegetables, fruits, aquatic products, edible oil, and eggs has declined. These shifts imply that the influence of past consumption on the food choices of Chinese rural households is gradually lessening, dietary habits are subtly weakening, and decision-making regarding current food consumption is becoming more assertive. In both time intervals, the habit-formation parameters for pork, beef, mutton, and poultry were not significant. Moreover, based on the estimations from the 2015–2018 sample, the habit formation parameter for egg was also insignificant. All the habit formation parameters that passed the significance tests were positive, with edible oil, grains, and vegetables exhibiting a greater degree of habit formation than other foods. This suggests that in recent years, Chinese rural households continue to maintain strong consumption habits for essential sustenance foods.

The preceding analysis reveals substantial temporal fluctuations in the extent of habit formation in food consumption. However, do these habit formation effect exhibit variations across different population segments? This study delves further into the nuances of habit formation in food consumption among rural households across diverse income and age brackets. Leveraging the World Bank’s middle-income poverty threshold, we segmented our sample into two income groups. Households with per capita income below this benchmark during the base period were classified as low-income, while those surpassing the benchmark were deemed as middle–high-income. The low-income and middle–high-income groups constituted 56.57% and 43.43% of the total sample, respectively.

For age group classification, we adhered to the United Nations World Health Organization’s age segmentation norms: individuals under 45 years are classified as youth, those between 45 and 60 years as middle-aged, and those over 60 years as elderly. We stratified households based on the age of the household head in the base year of 2012: those under 45 were categorized into the youth group and those above 45 into the middle-aged and elderly group. The youth group and the middle-aged and elderly group accounted for 40.03% and 59.97% of the total sample, respectively.

Table 4 presents the estimated degrees of habit formation in food consumption across distinct income groups. All habit formation parameters that passed the significance tests are positive, underscoring the entrenched habit formation traits in food consumption among Chinese rural residents. Furthermore, remarkable disparities existed in the habit formation parameters across different income groups. In the low-income group demonstrating the habit formation effect, legumes, vegetables, and edible oil demonstrate a stronger degree of habit formation, followed by grains and fruits. Aquatic products, eggs, and pork exhibit lesser habit formation intensity, and the habit formation parameters for beef, mutton, and poultry are statistically insignificant. For the middle–high-income group, the habit formation effect manifests in grains, vegetables, fruits, aquatic products, and edible oil, of which only grains and aquatic products possess a stronger degree of habit formation than the low-income group. This suggests that the middle–high-income group’s food consumption is less swayed by the preceding period’s food consumption status, particularly for animal-based foods, where only aquatic products display a habit formation effect.

Table 4 also showcases the estimated degrees of habit formation in food consumption segmented by age group. Excluding meat and poultry, the habit formation parameters for food consumption among both youth and middle-aged and elderly groups are significantly positive. This signifies that prior food consumption has a substantial promotional effect on the food consumption behaviors of various age groups. In alignment with the full-sample estimation outcomes, different age groups exhibit a stronger consumption habit for traditional food categories such as grains, vegetables, and edible oil than other food categories. Notable discrepancies in habits across age groups include the following: young farmers demonstrate a stronger habit formation intensity for grains, vegetables, fruits, pork, eggs, and edible oils than the middle-aged and elderly group, while the latter group manifests a more robust consumption habit for legumes and aquatic products. These observations emphasize that China’s rural youth persist in maintaining the legacy of dietary traditions.

### 3.2. Elasticity Estimation Results

In an endeavor to understand the evolving characteristics of food consumption demand among Chinese rural residents under the influence of habit formation, we computed the income elasticity of demand for various food categories. The second and third columns of Table 5 provide estimates derived from the dynamic Almost Ideal Demand System (AIDS) model, while the fourth and fifth columns offer estimates predicated on the static AIDS model.

As per the data in Table 5, the income elasticity for all food types is significantly positive. This suggests that with an upswing in income, future consumption across all food categories for Chinese rural households will correspondingly increase. More specifically, pork exhibits the smallest income elasticity, falling below 0.07. For commodities such as grains, vegetables, fruits, beef and mutton, poultry, aquatic products, and eggs, the income elasticity fluctuates between 0.07 and 0.08. The income elasticity of demand for legumes and edible oils exceeds 0.08, indicating a heightened sensitivity in consumption volumes in response to income fluctuations.

If we disregard the effect of habit formation in food consumption, the resulting income elasticity for beef and mutton, poultry, and aquatic products surpasses 0.1. This means that a 1% increase in income engenders a rise in consumption exceeding 0.1%. The income elasticity for other food categories is relatively lower. Compared to estimates factoring in habit formation, this approach undervalues the income elasticity for grains, legumes, and edible oils and overvalues that for vegetables, fruits, and animal foods. It is clear that considering the habit formation effect on food consumption leads to substantial shifts in the outlined food demand traits. Upon controlling for the habit formation effect, a notable characteristic emerges: the disparities in income elasticity among different food types markedly contract. As income increases, rural residents do not show a predilection towards amplifying their consumption of animal foods, but rather exhibit a balanced augmentation across all food types.

Table 6 documents the estimates for the nutritional demand elasticity of Chinese rural residents, with columns 2–3 based on the dynamic AIDS model and columns 4–5 on the static AIDS model. When considering habit formation in food consumption, the income elasticity for energy, protein, fat, and carbohydrates is significant, with elasticity values ranging from 0.073 to 0.081. Among these, fat exhibits the highest income elasticity, signaling the need for circumspection regarding potential health threats from excessive fat intake accompanying income growth. Neglecting habit formation in food consumption leads to an overestimation of protein’s income elasticity and an underestimation of that for energy, fat, and carbohydrates among Chinese rural residents. While protein, fat, and carbohydrates all serve as energy nutrients capable of providing energy to the body, protein primarily aids in cell and tissue growth and repair, only supplying energy when carbohydrate intake is insufficient. Fat intake is generally stored directly as fat, contributing to weight gain. Therefore, underestimating the income elasticity of fat and carbohydrates may result in underestimations of the obesity risk index.

### 3.3. Robustness Check

**Figure A1:**
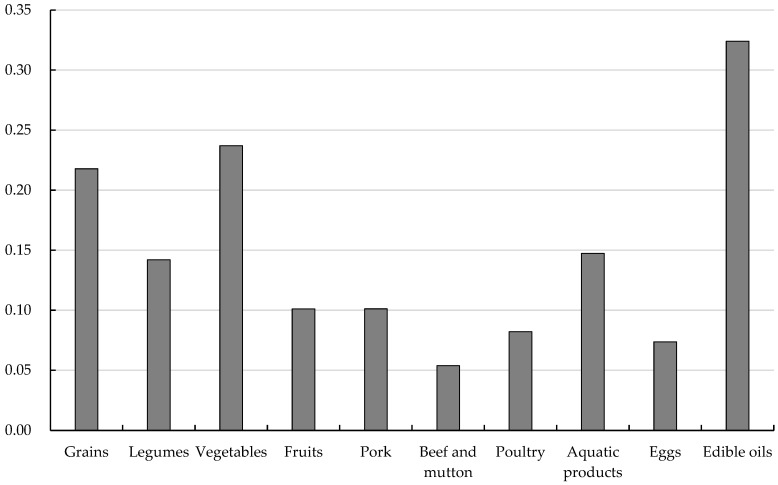
Estimation results for habit formation parameters from CHNS data.

To validate the robustness of the preceding findings, we used individual-level data from the China Health and Nutrition Survey (CHNS) spanning 2004 to 2011, enabling us to delve into the habit formation characteristics in food consumption amongst Chinese rural residents. The results derived from the CHNS data revealed that all ten food categories under scrutiny display significant habit-forming tendencies. The intensity of habit formation, in descending order, is as follows: edible oils, vegetables, grains, aquatic products, legumes, pork, fruits, poultry, eggs, and beef and mutton. Specific parameters associated with habit formation can be observed in Figure A1.

When juxtaposed with the research findings based on the household-level Survey for Agriculture and Village Economy (SAVE) data, we observed that edible oils, vegetables, and grains persist as the food categories with the strongest habit formation propensities. The habit-forming tendencies for poultry and beef and mutton remain relatively modest. However, a notable divergence is the increased habit formation intensity associated with the consumption of meat. This divergence could stem from differences in several underlying factors, including the diversity of provinces within the sample, granularity of the data, and the specific years from which the data were sourced.

Moreover, compared to the individual-level data of CHNS, studies focusing on household-level food consumption tend to mask disparities in individual consumption habits to some extent. Consequently, the degree of significance associated with habit formation estimates derived from SAVE data is marginally less than that inferred from the CHNS data. Notwithstanding the CHNS data, the core dataset employed in this study, the SAVE data, is updated up to 2018 and thus provides a more contemporaneous reflection of the food consumption patterns of residents, thereby enhancing its utility as a point of reference. Even when the unit of analysis is shifted to the household level, the impact of habit formation on food consumption remains discernable.

## 4. Discussion

The dietary behavior of China’s rural inhabitants exhibits a substantial habit formation effect, which manifests distinct variations across food types, over time, and between demographic groups. If the habit formation effect is ignored, it will amplify the differences in the income elasticity of various foods, underestimating the income elasticity of grains, legumes, and oils, while overestimating the income elasticity of vegetables, fruits and all animal foods. The habit formation effect of food consumption will further affect the nutritional demand elasticity. The income elasticity of energy, fat and carbohydrates based on the dynamic model is significantly higher than that based on the static model. Ignoring the habit formation effect of food consumption will lead to inaccurate characterization of food consumption demand.

The habit formation parameters for all food categories are notably positive, except for beef, mutton, and poultry. This is especially true for essential food items such as edible oils, grains, and vegetables. Edible oils are the main source of fat intake, which means that avoiding obesity by reducing one’s oil intake will not achieve the desired effect in the short term. However, over time, the propensity for habit formation in food consumption among rural residents has shown a declining trend and food consumption is less influenced by previous consumption. This trend indicates that as socio-economic development advances and food accessibility improves, the dietary habits of rural dwellers are undergoing significant transformations. The propensity to cling to traditional taste preferences is diminishing, suggesting that food producers should cater to a diversifying and personalized food market.

Analyzing by group, Chinese rural families with a low income and young families exhibit a stronger tendency toward habit formation in food consumption. Specifically, low-income families demonstrate a greater adherence to the consumption of legumes, vegetables, fruits, pork, eggs, and edible oils compared to their middle–high-income counterparts. Similarly, younger families show a stronger inclination for grains, vegetables, fruits, pork, eggs, and edible oils compared to middle-aged and elderly families. These findings imply that during the process of rural revitalization, targeted health dietary interventions are particularly necessary for low-income and younger demographics, fostering healthy food preferences early as income levels increase and individuals age. Attention should be paid to health problems such as obesity caused by excessive intake of edible oils.

When taking into account the habitual nature of food consumption, it is observed that rural residents increase their consumption of all types of food as their income rises, albeit at a modest rate. Interestingly, rather than favoring an increase in animal-based foods with a rising income, rural residents display a balanced growth pattern across all food types. This contradicts earlier research that reported a higher income elasticity for animal-derived foods [2,47,48]. However, it aligns with the findings of Hovhannisyan and Bozic, Hovhannisyan et al., and Hovhannisyan and Shanoyan, which indicate that the income elasticities across various food categories are relatively similar [31,49,50]. This suggests that income has a diminishing influence on the evolution of food consumption patterns, with habit formation playing a key role in maintaining a stable dietary structure. Although short-term price adjustments can marginally influence food consumption and nutritional intake behaviors [51], altering dietary habits is a long-term endeavor, highlighting the importance of sustained dietary intervention policies. Consequently, in the context of habit formation, it is inadequate to modify food consumption patterns solely through price adjustments, availability of food, and other similar measures. This also sheds light on why achieving weight loss through dietary changes can be particularly challenging in the short term.

If one considers the impact of habit formation on food consumption, it is found that the intake of energy, fat, and carbohydrates among rural residents will increase more substantially with rising incomes. This suggests that concurrent with changes in production and lifestyle, the transition of rural residents’ dietary patterns from low intake, high expenditure to high intake, low expenditure will accelerate. This shift could exacerbate issues of nutritional excess and obesity, potentially leading to a surge in chronic diseases such as diabetes and hypertension, and result in considerable socio-economic implications. Therefore, multiple measures should be implemented to guide residents towards healthy dietary practices, gradually improving past unhealthy food behaviors and dietary structures. Innovative methods of disseminating healthy eating knowledge should be explored, enhancing long-term promotion and guidance of healthy, scientific diets, and integrate nutritional and health concepts into all stages of food production, processing, cooking, and eating to cultivate a healthier food consumption paradigm and lifestyle philosophy among rural residents.

It should be noted that our study has several limitations. Based on this study’s results, adjusting dietary habits emerges as one viable strategy to optimize residents’ dietary structure. The development of policy interventions to modify dietary habits will be a focal point in our future research. However, research on the dissemination of information about food consumption remains insufficient. There is a pressing need for more profound studies to refine information optimization design [52]. This includes tailoring content for diverse groups and strategizing on how to effectively impart nutritional knowledge, which are critical for making information dissemination a more potent catalyst for transforming dietary habits. Furthermore, while the SAVE dataset comprises production data, it does not distinguish between the proportions of self-consumption and gifting. Consequently, this study’s quantification of rural residents’ food consumption includes only purchased food. Nonetheless, self-produced food is also a significant source of food consumption for rural inhabitants.

## 5. Conclusions

This research reveals that food consumption among China’s rural residents exhibited a pronounced habit formation effect, with essential survival foods like edible oils, grains, and vegetables showing a heightened degree of habit formation. With the passage of time, this habit formation tendency across all food categories has shown signs of decline. Furthermore, it has been observed that low-income and younger demographics display more entrenched food consumption habits compared to their middle–high-income, and middle-aged or elderly counterparts. The existence of such a habit formation effect significantly influences the estimations of food demand elasticity and nutritional demand elasticity. When accounting for these habit formation characteristics, balanced growth in the consumption of all food types is projected as income increases, thereby diminishing the explanatory power of income over shifts in food consumption structures. Overlooking these habit formation effect in food consumption could lead to an underestimation of income elasticity for energy, fats, and carbohydrates, and an overestimation for proteins. Therefore, in order to conduct a comprehensive analysis of food consumption demand, it is paramount to factor in dietary habits, while concurrently acknowledging variations across different demographic groups and time periods. This approach will aid in deriving more precise research conclusions and enhancing the accuracy of extrapolated findings related to nutritional health.

## Figures and Tables

**Figure 2:**
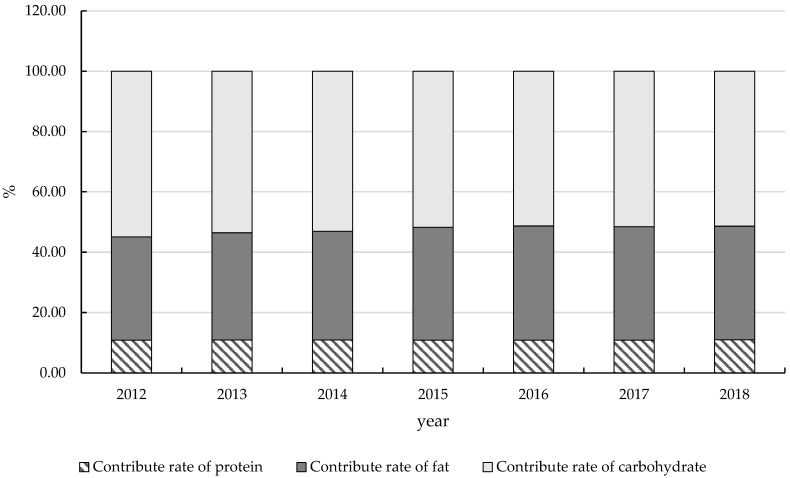
Energy contribution ratios in rural China.

**Table 1:** Food intake in the sample data (unit: kg/year).

Food Items	2012	2013	2014	2015	2016	2017	2018	2012–2018 Growth (%)
Grains	272.5	258.8	254.8	239.1	231.3	230.6	224.5	−17.6
Legumes	17.5	20.0	21.6	21.9	21.7	21.1	21.7	24.2
Vegetables	124.3	132.7	140.2	137.3	138.9	135.8	138.7	11.5
Fruits	49.9	49.6	49.8	53.2	52.2	52.4	51.1	2.3
Pork	42.2	45.4	45.6	45.5	44.1	44.5	44.7	5.8
Beef and mutton	3.5	4.3	5.0	4.8	5.2	5.3	5.5	54.7
Poultry	11.4	10.6	10.9	10.5	10.5	9.7	9.3	−18.9
Aquatic products	14.6	14.3	14.7	14.6	15.2	14.7	15.5	5.8
Eggs	23.0	23.8	23.5	24.1	25.3	25.5	26.3	14.3
Edible oils	37.6	37.7	38.7	39.4	39.3	38.3	37.1	−1.3

**Table 2:** Summary statistics for major variables.

Variables	Unit	Mean	S.D.
Annual real income of the household	CNY	45,015.98	46,364.12
Annual real expenditure on food purchases by the household	CNY	4660.04	2604.11
Actual price of grains	CNY/kg	5.71	3.19
Actual price of legumes	CNY/kg	8.56	4.31
Actual price of vegetables	CNY/kg	5.29	3.47
Actual price of fruits	CNY/kg	6.99	4.48
Actual price of pork	CNY/kg	22.96	6.35
Actual price of beef and mutton	CNY/kg	66.45	29.72
Actual price of poultry	CNY/kg	19.41	8.41
Actual price of aquatic products	CNY/kg	16.10	5.58
Actual price of eggs	CNY/kg	11.17	4.65
Actual price of edible oil	CNY/kg	14.52	5.63
Number of household members	persons/household	3.57	1.41
Gender of the head of household, male = 1, female = 0	/	0.94	0.23
Actual age of the head of household	/	51.08	10.17
Number of years of education of the head of the household	/	8.03	2.29
Proportion of working population to the number of household members	%	67.00	31.16
Sample is in the eastern region = 1, otherwise = 0	/	0.53	0.50
Sample is in the central region = 1, otherwise = 0	/	0.33	0.47
Sample is in the western region = 1, otherwise = 0	/	0.13	0.34
Actual year of the survey	/	2015.00	2.00

**Table 3:** Estimation results for habit formation parameters.

Variables	Grains	Legumes	Vegetables	Fruits	Pork	Beef and Mutton	Poultry	Aquatic Products	Eggs	Edible Oils
$q_{it-1}$	0.100 ***	0.065 ***	0.095 ***	0.063 ***	0.026 *	0.007	0.016	0.058 ***	0.051 ***	0.103 ***
	(0.013)	(0.015)	(0.013)	(0.015)	(0.015)	(0.016)	(0.019)	(0.015)	(0.015)	(0.013)
Income and price	YES	YES	YES	YES	YES	YES	YES	YES	YES	YES
Demographics	YES	YES	YES	YES	YES	YES	YES	YES	YES	YES
Region	YES	YES	YES	YES	YES	YES	YES	YES	YES	YES
Time	YES	YES	YES	YES	YES	YES	YES	YES	YES	YES
Observations	3882	3882	3882	3882	3882	3882	3882	3882	3882	3882

Notes: Standard errors in parentheses; * *p* < 0.10, *** *p* < 0.01.

**Table 4:** Group differences in the habit formation effect of food consumption.

Food Items	Low-Income Group		Middle–High-Income Group		Youth Group		Middle-Aged and Elderly Group	
	Habit Formation Parameters	S.D.	Habit Formation Parameters	S.D.	Habit Formation Parameters	S.D.	Habit Formation Parameters	S.D.
Grains	0.096 ***	(0.018)	0.115 ***	(0.022)	0.168 ***	(0.018)	0.096 ***	(0.017)
Legumes	0.100 ***	(0.030)	0.038	(0.024)	0.072 ***	(0.023)	0.078 ***	(0.019)
Vegetables	0.110 ***	(0.018)	0.093 ***	(0.021)	0.152 ***	(0.020)	0.091 ***	(0.017)
Fruits	0.091 ***	(0.020)	0.046 **	(0.021)	0.104 ***	(0.022)	0.059 ***	(0.019)
Pork	0.036 *	(0.020)	0.019	(0.024)	0.045 *	(0.025)	0.029	(0.019)
Beef and mutton	−0.001	(0.021)	0.006	(0.025)	0.001	(0.024)	0.008	(0.023)
Poultry	0.006	(0.021)	0.013	(0.051)	0.040	(0.025)	0.011	(0.024)
Aquatic products	0.059 ***	(0.021)	0.061 ***	(0.023)	0.060 **	(0.024)	0.069 ***	(0.021)
Eggs	0.059 ***	(0.019)	0.036	(0.024)	0.089 ***	(0.022)	0.045 **	(0.020)
Edible oils	0.141 ***	(0.016)	0.102 ***	(0.020)	0.163 ***	(0.020)	0.112 ***	(0.017)
Observations	2196		1686		1554		2328	

Notes: Standard errors in parentheses; * *p* < 0.10, ** *p* < 0.05, *** *p* < 0.01.

**Table 5:** Food elasticity estimation results.

Food Items	Considering the Habit Formation Effect		Not Considering the Habit Formation Effect	
	Income Elasticity	S.D.	Income Elasticity	S.D.
Grains	0.073 **	(0.037)	0.046 **	(0.021)
Legumes	0.081 **	(0.041)	0.073 **	(0.033)
Vegetables	0.073 **	(0.037)	0.089 **	(0.040)
Fruits	0.073 **	(0.037)	0.074 **	(0.034)
Pork	0.068 **	(0.034)	0.092 **	(0.042)
Beef and mutton	0.071 **	(0.036)	0.329 **	(0.149)
Poultry	0.074 *	(0.038)	0.112 **	(0.051)
Aquatic products	0.076 **	(0.038)	0.105 **	(0.048)
Eggs	0.072 **	(0.036)	0.092 **	(0.042)
Edible oils	0.089 *	(0.047)	0.062 **	(0.028)

Notes: Standard errors in parentheses; * *p* < 0.10, ** *p* < 0.05.

**Table 6:** Nutrient elasticity estimation results.

Nutrient Items	Considering the Habit Formation Effect		Not Considering the Habit Formation Effect	
	Income Elasticity	S.D.	Income Elasticity	S.D.
Energy	0.077 **	(0.039)	0.062 **	(0.028)
Protein	0.073 **	(0.037)	0.074 **	(0.034)
Fat	0.081 **	(0.041)	0.073 **	(0.033)
Carbohydrate	0.073 **	(0.037)	0.059 **	(0.027)

Notes: Standard errors in parentheses; ** *p* < 0.05.

## Data Availability

The data presented in this study are available upon request from the corresponding author.

## Appendix A

**Table A1:** Dynamic AIDS model estimation results.

	Grains	Legumes	Vegetables	Fruits	Pork	Beef and Mutton	Poultry	Aquatic Products	Eggs	Edible Oils
$ln{\hat{p}}_{1}$	0.008									
	(0.028)									
$ln{\hat{p}}_{2}$	0.000	0.002								
	(0.019)	(0.013)								
$ln{\hat{p}}_{3}$	−0.002	0.003	0.003							
	(0.007)	(0.005)	(0.003)							
$ln{\hat{p}}_{4}$	−0.002	0.000	0.000	0.003						
	(0.002)	(0.001)	(0.002)	(0.002)						
$ln{\hat{p}}_{5}$	−0.001	−0.004	−0.002	0.001	0.022					
	(0.042)	(0.030)	(0.011)	(0.005)	(0.066)					
$ln{\hat{p}}_{6}$	0.004	0.000	0.001	0.002	−0.016	0.015				
	(0.055)	(0.039)	(0.013)	(0.003)	(0.084)	(0.111)				
$ln{\hat{p}}_{7}$	−0.003	−0.003	−0.004	0.000	0.004	−0.002	0.001			
	(0.025)	(0.018)	(0.007)	(0.003)	(0.039)	(0.051)	(0.023)			
$ln{\hat{p}}_{8}$	−0.008	−0.003	0.003	−0.001	0.001	−0.005	−0.001	0.006		
	(0.010)	(0.007)	(0.003)	(0.002)	(0.016)	(0.020)	(0.010)	(0.005)		
$ln{\hat{p}}_{9}$	0.002	0.000	−0.003	0.000	−0.005	0.000	−0.003	0.000	0.004	
	(0.014)	(0.010)	(0.004)	(0.001)	(0.022)	(0.029)	(0.014)	(0.006)	(0.008)	
$ln{\hat{p}}_{10}$	0.001	0.007	0.000	−0.002	0.002	0.003	0.012	0.009	0.004	−0.036
	(0.115)	(0.081)	(0.027)	(0.005)	(0.175)	(0.233)	(0.107)	(0.043)	(0.061)	(0.489)
$ln\left(\frac{{\hat{x}}_{t}}{a\left({\hat{p}}_{t}\right)}\right)$	0.006	0.004	0.001	0.000	−0.009	0.012	0.006	0.002	0.003	−0.026
	(0.006)	(0.003)	(0.003)	(0.003)	(0.010)	(0.008)	(0.007)	(0.003)	(0.003)	(0.021)
Size	0.001	−0.001	0.000	0.000	0.000	−0.002	0.000	0.000	0.000	0.002
	(0.002)	(0.000)	(0.001)	(0.001)	(0.002)	(0.002)	(0.001)	(0.001)	(0.001)	(0.006)
Gender	−0.002	0.000	0.002	0.000	0.001	0.000	0.001	0.000	0.001	−0.004
	(0.012)	(0.002)	(0.005)	(0.003)	(0.010)	(0.013)	(0.005)	(0.003)	(0.003)	(0.030)
Age	0.000	0.000	0.000	0.000	0.000	0.000	0.000	0.000	0.000	0.000
	(0.000)	(0.000)	(0.000)	(0.000)	(0.000)	(0.000)	(0.000)	(0.000)	(0.000)	(0.001)
Education	−0.001	0.000	0.000	0.000	0.000	0.000	0.000	0.000	0.000	0.000
	(0.001)	(0.000)	(0.001)	(0.000)	(0.001)	(0.001)	(0.001)	(0.000)	(0.000)	(0.003)
Proportion of Working Population	−0.001	0.000	0.002	0.001	0.006	−0.006	0.000	−0.002	−0.002	0.001
	(0.010)	(0.002)	(0.005)	(0.003)	(0.008)	(0.011)	(0.004)	(0.003)	(0.002)	(0.026)
Constant	−0.043	−0.043	0.005	−0.032	0.286	−0.228	0.031	0.001	−0.005	1.028
	(0.492)	(0.355)	(0.123)	(0.045)	(0.752)	(0.998)	(0.478)	(0.186)	(0.266)	(2.120)
Region	YES	YES	YES	YES	YES	YES	YES	YES	YES	YES
Time	YES	YES	YES	YES	YES	YES	YES	YES	YES	YES
